# Chondromodulin-1 in health, osteoarthritis, cancer, and heart disease

**DOI:** 10.1007/s00018-019-03225-y

**Published:** 2019-07-17

**Authors:** Sipin Zhu, Heng Qiu, Samuel Bennett, Vincent Kuek, Vicki Rosen, Huazi Xu, Jiake Xu

**Affiliations:** 1 Department of Orthopaedics, The Second Affiliated Hospital and Yuying Children’s Hospital of Wenzhou Medical University, Wenzhou, 325000 Zhejiang China; 2 Molecular Laboratory and the Division of Regenerative Biology, School of Biomedical Sciences, M Block, QEII Medical Centre, The University of Western Australia (M504), 35 Stirling Hwy, Perth, WA 6009 Australia; 3 Developmental Biology, Harvard School of Dental Medicine, Boston, MA 02115 USA

**Keywords:** Embryo, Heart, Fertility, Bone, Immune, Tumour

## Abstract

The human chondromodulin-1 (Chm-1, Chm-I, CNMD, or Lect1) gene encodes a 334 amino acid type II transmembrane glycoprotein protein with characteristics of a furin cleavage site and a putative glycosylation site. Chm-1 is expressed most predominantly in healthy and developing avascular cartilage, and healthy cardiac valves. Chm-1 plays a vital role during endochondral ossification by the regulation of angiogenesis. The anti-angiogenic and chondrogenic properties of Chm-1 are attributed to its role in tissue development, homeostasis, repair and regeneration, and disease prevention. Chm-1 promotes chondrocyte differentiation, and is regulated by versatile transcription factors, such as Sox9, Sp3, YY1, p300, Pax1, and Nkx3.2. Decreased expression of Chm-1 is implicated in the onset and progression of osteoarthritis and infective endocarditis. Chm-1 appears to attenuate osteoarthritis progression by inhibiting catabolic activity, and to mediate anti-inflammatory effects. In this review, we present the molecular structure and expression profiling of Chm-1. In addition, we bring a summary to the potential role of Chm-1 in cartilage development and homeostasis, osteoarthritis onset and progression, and to the pathogenic role of Chm-1 in infective endocarditis and cancers. To date, knowledge of the Chm-1 receptor, cellular signalling, and the molecular mechanisms of Chm-1 is rudimentary. Advancing our understanding the role of Chm-1 and its mechanisms of action will pave the way for the development of Chm-1 as a therapeutic target for the treatment of diseases, such as osteoarthritis, infective endocarditis, and cancer, and for potential tissue regenerative bioengineering applications.

## Introduction

The chondromodulin-1 (Chm-1, Chm-I, CNMD, or Lect1) gene was first cloned from foetal bovine cartilage as a 25 kDa glycoprotein that could stimulate chondrocyte growth in the presence of basic fibroblast growth factor (FGF) [1]. Chm-1 has structural characteristics of a type II transmembrane glycoprotein with two cleaved portions: the N-terminus contains a surfactant protein referred to as a chondrosurfactant protein, and the C-terminus of the precursor protein consists of a 25 kDa mature protein referred to as Chm-1, which is able to stimulate chondrocyte growth and to inhibit angiogenesis [1–4]. During endochondral bone formation, Chm-1 is involved in the regulation of cartilage growth and vascular invasion prior to the transition of cartilage to bone [5–7]. The Chm-1 protein is mainly detected in the avascular region of pre-hypertrophic cartilage, and appears to prevent vascularization of cartilaginous anlagen during endochondral ossification [8, 9]. Chm-1 may be also involved in the pathogenesis of conditions characterized by neo-vascularization, such as infective endocarditis, and cancers [4, 10]. The aim of this article is to provide a current overview of the characterization of Chm-1, in terms of expression profiling, molecular structure, and biological function. Overall, Chm-1 appears to be involved in the regulation of tissue angiogenesis, cartilage development and homeostasis, and to be implicated in the onset and progression of diseases, such as osteoarthritis, infective endocarditis, and cancer.

## Molecular structure, gene expression, and function of Chm-1

**Fig. 1:**
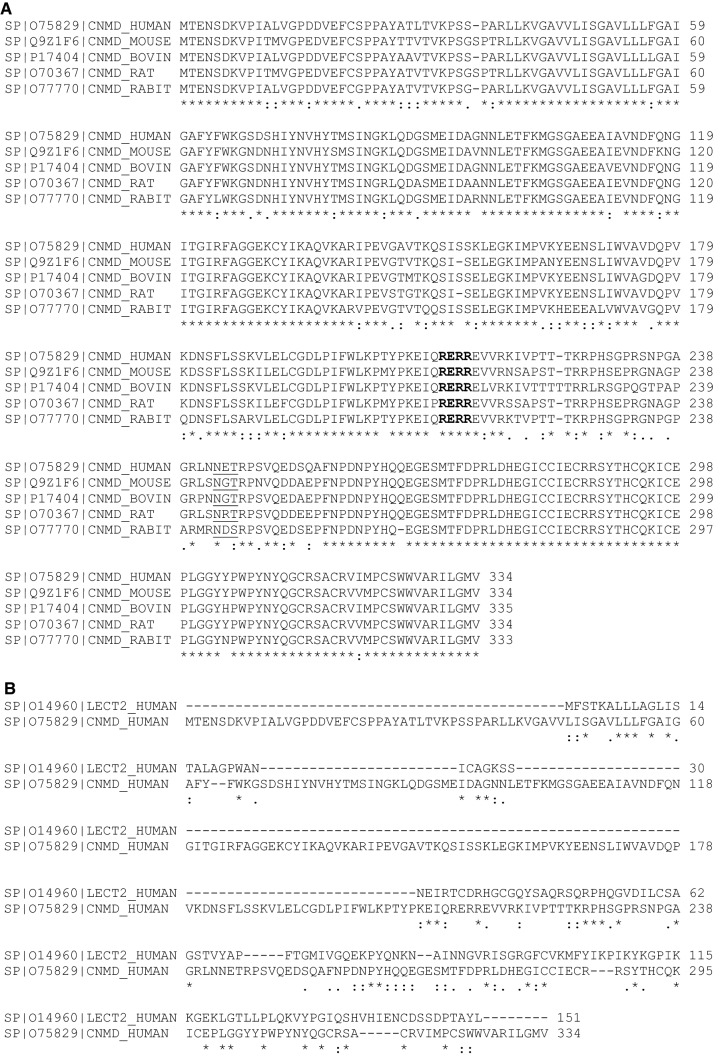
Multiple sequence alignment showing substantial identity among Chm-1 amino acid sequences in human, mouse, rat, bovine, and rabbit, with a consensus potential glycosylation site underlined, and a putative furin cleavage site (RERR) in bold font (**a**). Multiple sequence alignment showing limited homology between human Chm-1 (LECT1) and chondromodulin-II (LECT2) amino acid sequence at their C-terminal regions (**b**)

**Fig. 2:**
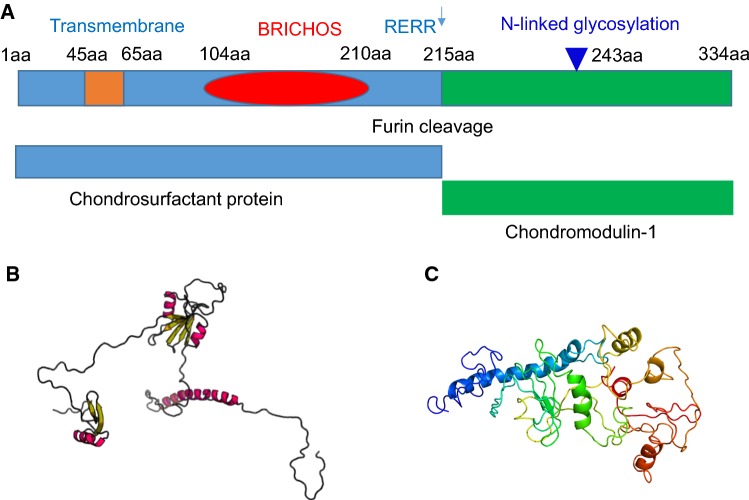
Molecular structure of Chm-1. Secondary structure of Chm-1 showing that it contains a transmembrane domain between amino acid residues 40–65, a furin cleavage site (RERR) at 215 amino acid residue, an N-linked glycosylation site (NET) at 243 amino acid residue, and a BRICHOS domain from 104–210 amino acid residues (**a**). Tertiary structure analysis reveals that it features a typical helical conformation based on the Phyre2 web portal for protein modelling (http://twitter.com/phyre2server) (**b**), and by RaptorX template-based protein structure modelling (http://raptorx.uchicago.edu/StructurePrediction) (**c**)

The human chondromodulin-1 gene (Chm-1, or Chm-I), also referred to as CNMD, leukocyte cell-derived chemotaxin 1, or Lect 1 is located on chromosome 13q14-21, and encodes a 334 amino acid type II transmembrane glycoprotein. Multiple sequence alignment indicates that inter-species sequence homology among human, mouse, rat, bovine and rabbit Chm-1 homologs is conserved, by approximately 90% amino acid sequence identity between species homologs (Fig. 1a). Chm-1 only shares limited sequence similarity with chondromodulin-II (or Lect2) at the C-terminus (Fig. 1b). Protein sequence analyses indicate that human Chm-1 contains a transmembrane domain between amino acid residues 40–65, a furin cleavage site (RERR) at amino acid residue 215, and an N-linked glycosylation site (NET) at amino acid residue 243 (Fig. 2a). The Chm-1 protein also consists of a BRICHOS domain from amino acid residues 104–210, that is referred to as lung surfactant protein C proprotein (proSP-C), although its biological activity is yet to be experimentally examined (Fig. 2a). Tertiary structure analysis reveals that the Chm-1 protein features a typical helical conformation, based on the Phyre2 protein modelling web portal, and by RaptorX template-based protein structure modelling, respectively (Fig. 2b, c) [11–13].

Further structural and functional analyses indicate that Chm-1 encodes a type II transmembrane glycoprotein, which was detected in two forms [8]. A 14 kDa species was generated by proteolytic cleavage at Asp37–Asp38 at the N-terminus, which appears to have little or no inhibitory anti-angiogenic activity, and to be located in the hypertrophic and calcified zones. By comparison, the intact 20–25 kDa species was predominantly detected in the avascular zones, including resting, proliferating, and pre-hypertrophic zones of developing long bones [8]. The intact Chm-1 protein is abundantly expressed in the inner meniscus of the knee, and appears to be an anti-angiogenic factor that is important for maintaining the avascularity of the inner meniscus by inhibiting endothelial cell proliferation [14]. During endochondral ossification, Chm-1 is expressed in the avascular zone of cartilage, but not calcifying cartilage, and appears to prevent vascular invasion during bone formation [2]. Chm-1 is sensitive to furin-mediated cleavage and the mature form of human Chm-1 is present in the cell culture supernatant, which is indicative of its secretory nature [15, 16]. Further, the C-terminus of the mature, secreted, intact form of Chm-1, exerts anti-angiogenic and anti-tumour activities, and bears similar function and homology to the C-terminal domain of the tenomodulin (TeM) glycoprotein [10]. Interestingly, chondromodulin-II (or Lect2), which shares limited sequence homology at the C-terminus with Chm-1, is a component protein of cartilage matrix that promotes chondrocyte proliferation during endochondral ossification [17].

Chm-1 mRNA is expressed most abundantly in avascular cartilage and cardiac valves, and has been shown to promote chondrocyte differentiation and to inhibit angiogenesis by endothelial cell-mediated tubule formation [1, 2, 4, 14]. Chm-1 is also expressed in the CD31(−) avascular mesenchyme and eyes [18, 19]. The differential expression pattern of Chm-1 mRNA and tenomodulin (TeM) mRNA was compared in mandibular condylar cartilage and tibial cartilage, with Chm-1 mRNA less abundant in mandibular condylar cartilage than in tibial as compared to TeM mRNA [20]. A number of studies have shown that its expression appears to be regulated by transcription factors Sox9, Sp3, histone modifiers (YY1 and p300), and Pax1 and Nk3 homeobox 2 (Nkx3.2) [21–24]. Chm-1 appears to maintain cartilage homeostasis by inhibiting hypoxia-inducible factor-2α (HIF-2α) induced catabolic activity, and may be a potential therapeutic target for the inhibition of cartilage degradation resulting from osteoarthritis [25].

**Fig. 3:**
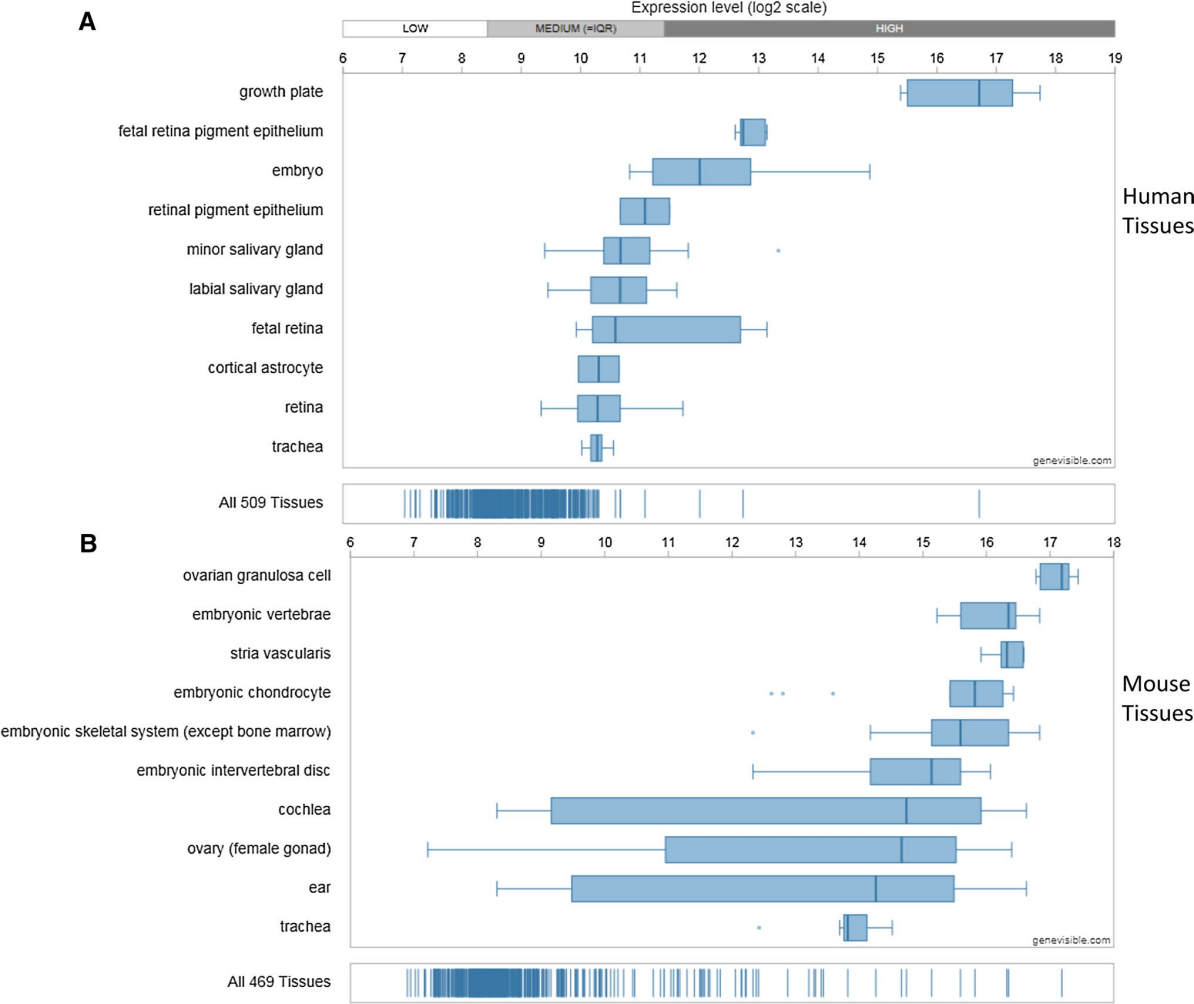
Expression analyses showing the Chm-1 expression in both human (**a**) and mouse (**b**) tissues, with 10 most highly expressed tissues for each species, performed by Genevisible^®^ (http://genevisible.com)

**Fig. 4:**
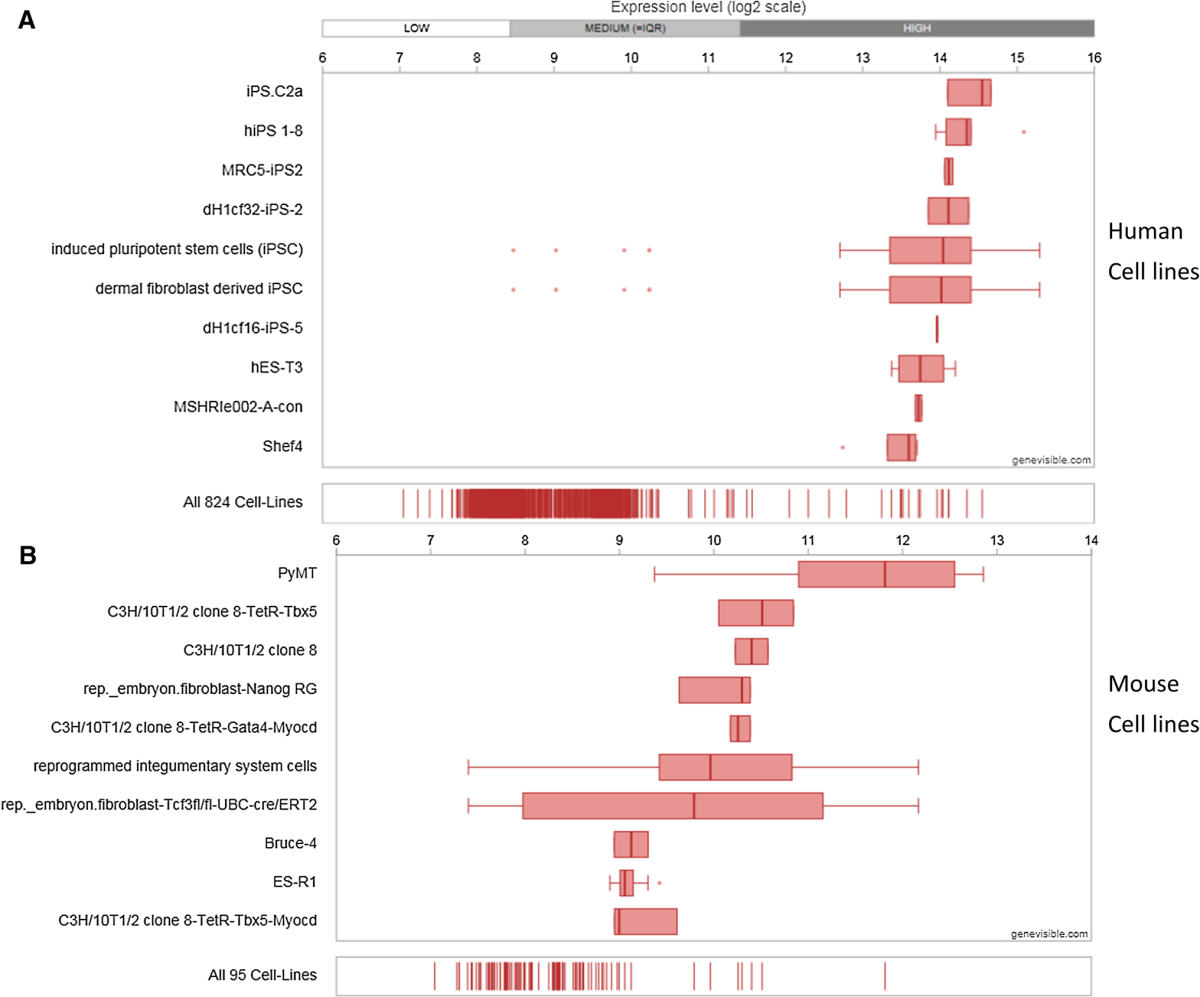
Expression analyses showing the Chm-1 expression in both human (**a**) and mouse (**b**) cell lines, with 10 most highly expressed cell lines for each species, performed by Genevisible^®^ (http://genevisible.com)

Utilising Genevisible^®^ gene profiling analyses, Chm-1 mRNA expression is detected in human and mouse, tissues and cell lines [26]. Chm-1 mRNA appears to be most abundantly expressed in human growth plate, human foetal retina pigment epithelium cells, and human embryo tissues, and mouse ovarian granulosa cell, mouse embryonic vertebrae, and mouse stria vascularis tissues (Fig. 3). In addition, Chm-1 mRNA is expressed in human and mouse cell lines. Chm-1 mRNA most abundantly expressed in the iPS.C2a, hiPS 1-8, and MRC5- iPS2 human cell lines, and the PyMT, C3H/10T1/2 clone 8-TetR-Tbx5, and C3H/10T1/2 clone 8 mouse cell lines (Fig. 4).

## The effect of Chm-1 in cartilage and osteoarthritis (OA) progression

Several lines of evidence suggest that Chm-1 is involved in the regulation of chondrocyte differentiation and cartilage homeostasis [25, 27–30]. In vitro findings suggest that Chm-1 promotes the growth of chondrocytes via an autocrine signalling mechanism in the presence of fibroblast growth factor 2 (FGF-2) [31]. Targeted disruption of the Chm-1 gene showed no obvious phenotypic abnormality of endochondral bone formation during embryogenesis or cartilage development during growth stages of Chm-1(−/−) mice [6, 30]. Interestingly, adult Chm-1(−/−) mice showed an increased bone mineral density phenotype, with decreased bone resorption, indicating that Chm-1 may also be an important factor for bone remodelling and metabolism [6]. Notably, Chm-1 gene knockout directly restricted ectopic cartilage regeneration in vivo and inhibited ectopic stability of regenerated cartilage in vitro, suggesting that Chm-1 plays a role in ectopic cartilage regeneration and cartilage homeostasis [30]. Since Chm-1 appears to be an important factor for the maintenance of cartilage homeostasis and bone metabolism, the effect of Chm-1 knockout on bone repair was tested [5]. Findings from this study indicate that the healing following bone fracture is delayed in Chm-1(−/−) mice, which appears to be the result of impaired periosteal chondrocyte differentiation and reduced cartilaginous callus formation during the healing process [5]. Collectively, these findings indicate that Chm-1 is an important factor for chondrocyte maturation and cartilage homeostasis, and could be involved in bone remodelling and repair.

Articular cartilage is an avascular tissue that is composed of extracellular matrix and sparsely populated by chondrocytes, thus providing a smooth, lubricated surface for the function of diarthrodial joints [32]. Osteoarthritis (OA) is a chronic joint disease characterized by cartilage destruction, subchondral bone sclerosis, and osteophyte formation, which appear to be accompanied by vascular invasion of the articular cartilage [33, 34]. Decreased expression of Chm-1 was found to be associated with the onset and progression of OA [35]. The loss of Chm-1 protein function may exacerbate OA progression due to the loss of anti-angiogenic activity of Chm-1, and the subsequent vascular invasion of articular cartilage, which may be accompanied by chondrocyte hypertrophy [25, 35]. HIF-2α appears to cause OA cartilage destruction via matrix degradation, chondrocyte apoptosis, and an angiogenic signalling cascade [25, 36]. Induced expression of Chm-1 was found to mitigate OA progression via attenuating HIF-2α nuclear translocation and transcriptional activity [25]. In addition, Chm-1 overexpression appears to reduce tumour necrosis factor alpha (TNF-α) induced chondrocyte hypertrophy, and in vivo intra-articular administration of LV-Chm-1 appears to slow OA progression [25]. Further, thinning of the articular cartilage and the appearance of a vascular channel resulting from immobilization-induced OA cartilage degradation is associated with reduced expression of Chm-1, and increased expression of HIF-1α and vascular endothelial growth factor (VEGF) [37]. Chm-1 appears to stabilize the chondrocyte phenotype by supporting chondrogenesis, whilst inhibiting chondrocyte hypertrophy and aberrant endochondral ossification during cartilage tissue repair, which is accompanied by the upregulation of the cell cycle inhibitor p21WAF1/Cip1 [29]. Collectively, these findings suggest that Chm-1 plays a role in the regulation of chondrocyte differentiation and maturation, in the protection from the onset and progression of osteoarthritis, and is involved in the regulation of cartilage repair.

Recently, the significance of Chm-1 expression associated with angiogenesis during the pathogenesis of temporomandibular joint (TMJ) osteoarthritis was investigated [38]. Chm-1 protein expression was observed in the proliferative and hypertrophic zone of mandibular condylar cartilage, and chondrocyte-like cells in the TMJ disc by immunohistochemistry analyses; which is indicative of a role of Chm-1 in the regulation of TMJ remodelling via preventing blood vessel invasion of the condylar cartilage and by maintaining the integrity of the condylar cartilage and TMJ disc [28]. Lack of expression of Chm-1 in deep hypertrophic chondrocytes may result in vascular invasion of the condylar cartilage and lead to TMJ osteoarthritis [34].

The anti-angiogenic and chondrogenic properties of Chm-1 have been utilised for potential bioengineering applications. Interestingly, the inhibition of vascular ingrowth by the upregulation of Chm-1 on platelet-rich plasma (PRP)/hydrogel composites, provided an improved microenvironment for the regeneration of hyaline cartilage [39]. Further, the chondrogenic phenotype of marrow mesenchymal stem cell (MSC)-cartilage grafts may be stabilized by Chm-1 gene-modification leading to improved success of MSC-cartilage grafts; and the chondrogenesis and cartilage regenerative potential of MSCs may be enhanced by Chm-1 gene transfection [40, 41]. Taken together, these results indicate that the genetic modulation of Chm-1 in relation to chondrogenesis and cartilage regeneration may be a potential therapeutic strategy for osteoarthritis treatment by the improvement of regenerative tissue engineering applications.

## The role of Chm-1 in cancer and heart disease

The role of Chm-1 in cancers is largely unknown. The anti-angiogenic properties of Chm-1 are postulated to inhibit tumour growth, propagation, and metastasis, indicating that loss of Chm-1 function may induce tumorigenesis [42]. Significantly, Chm-1 appears to be implicated in the pathogenesis of the two most common childhood bone tumours, osteosarcoma and Ewing’s sarcoma (ES) [42, 43]. Chm-1 has been shown to inhibit the growth and proliferation of human osteosarcoma cells in vivo, and to inhibit the invasion and migration of human osteosarcoma cells in vitro [42]. Further research is necessary to investigate the properties and mechanisms by which Chm-1 could inhibit osteosarcoma tumorigenesis, and to develop the therapeutic potential of Chm-1 for the treatment of osteosarcoma. Chm-1 was found to be overexpressed in ES (the second most common childhood bone malignancy), which appears to be regulated by oncogenic fusion protein chimeric transcription factor, EWS-FLI1 [43]. Chm-1 could enhance the invasive potential of ES cells in vitro, and may promote ES lung metastases in vivo via the regulation of matrix metalloproteinase 9 (MMP9) expression [43]. Chm-1 appears to promote the malignancy of ES by maintaining its immature chondrocytic phenotype [43]. Further, although Chm-1 appears to inhibit endothelial differentiation in vitro and in vivo, no difference in angiogenesis was observed for in vivo tumour samples, suggesting that modulation of angiogenesis by Chm-1 does not appear to enhance ES metastasis [43]. As an enhancer of ES malignancy, Chm-1 is a prime therapeutic target warranting further investigation. Chm-1 directed allorestricted T-cell receptor (TCR) transgenic CD8+ T cells have been developed and demonstrate the capacity to specifically inhibit ES growth in vitro and in vivo [44]. Moreover, clinical research shows that Chm-1-specific allorestricted T-cell receptor (TCR) transgenic T cells home to bone marrow metastases and may cause partial disease regression without graft versus host disease (GvHD) in ES patients, indicating the need for future clinical trials [45]. Taken together, these findings suggest that the role of Chm-1 in the pathogenesis of ES appears to be pleiotropic, indicating the need for further investigation, and development of the potential of Chm-1 as a therapeutic target for ES [43]. Additionally, Chm-1 expression was shown to be downregulated in gastric cancer, and Chm-1 appears to be a potential tumour suppressor, that may also be an important biomarker for the treatment and prognosis of gastric cancer [46]. Chm-1 is also differentially expressed between benign and malignant (thyroid carcinomas) tumours of patients with multiple endocrine neoplasia (MEN), thus providing molecular evidence for the skeletal abnormalities and malignancy of different forms of MEN [47]. Chm-1 expression was also detected in lacuna cells and neoplastic myoepithelial cells during the pathogenesis of pleomorphic adenoma, which is indicative of its involvement in the hypo-vascularity and chondroid formation of pleomorphic adenoma [48]. At the cellular level, Chm-1 appears to be capable of exhibiting a dual role by promoting cell proliferation and suppressing the growth of tumour cells in a dose-dependent relationship [49]. Low concentrations of Chm-1 appear to promote osteoblastic-cell growth, whereas higher concentrations of Chm-1 suppressed the growth and migration of tumour cells, such as human cervical cancer (HeLa) cells and human neuroblastoma (SH-SY5Y) cells, and inhibited the proliferation and angiogenesis of human umbilical vein endothelial cells (HUVECs) [49]. Reports suggest that the anti-angiogenic properties of Chm-1 may contribute to the inhibitory effect of tumour progression [50, 51]. Chm-1 exerts a direct anti-tumour effect by inhibiting the STAT signalling pathway [50]. Mechanistic studies revealed that Chm-1 can induce human nasopharyngeal carcinoma (NPC) cell apoptosis by inhibiting the formation of the cell surface-associated endoplasmic reticulum chaperone glucose-regulated protein 78 (GRP78)-phosphatidylinositol 3-kinase-protein kinase B (PI3K-AKT) signalling complex [52]. Chm-1 also appears to be capable of inhibiting the growth of breast cancer cells by modulating the expression levels of cell cycle associated genes, and thus may have potential clinical applications for the treatment of breast cancer [53]. Collectively, these findings suggest a role for Chm-1 in the pathogenesis of a variety of cancers, which appears to be related to the regulation of chondrocytic differentiation and anti-angiogenic properties of Chm-1, thus indicating the need for further investigation. The cellular and molecular signalling pathways involved in cancer pathogenesis of Chm-1 are largely unknown and require further testing. The role of Chm-1 in cancer pathogenesis warrants further investigation to develop its potential as a therapeutic target for cancer treatment.

Inflammation-induced neo-vascularization is a pathogenic hallmark in the development of infective endocarditis (IE). Chm-1 expression is downregulated in valvular tissues at the early phase of IE, which might lead to leaflet vascularization and the progression of endocarditis, likely by a combination of the reduced anti-angiogenic resistance of leaflets and the immune-mediated inflammatory response resulting from decreased Chm-1 levels [54, 55]. The anti-angiogenic properties of Chm-1 that protect cardiac valves from pathological vascularization, may be attributed to the regulation of angiogenesis and matrix metalloproteinases [56, 57]. Further, hypoxia-associated reduced expression of Chm-1 might also be a factor for mid-to-late valve disease progression [58]. During implantation, Chm-1 was shown to have an inhibitory effect on trophoblast migration and invasion, which implies that Chm-1 may affect the regulation of tissue differentiation and angiogenesis during embryonic development [59]. Further studies are required to advance our understanding of the role of Chm-1 in the development of infective endocarditis, and embryogenesis.

## Chm-1 receptor and signalling

The receptor for Chm-1 is not known. In a study of human nasopharyngeal carcinoma cells (NPC), Chm-1 was found to induce NPC cell apoptosis by inhibiting the formation of the cell surface-associated endoplasmic reticulum chaperone GRP78-PI3K-AKT signalling complex [52]. The downstream effects of PI3K/AKT signalling might affect the mTOR network, responsible for cell survival. The direct tumour cell suppressing effect of Chm-1 appears to be mediated by its inhibition of the STAT signalling cascade [50]. Further studies are required to identify and characterize the Chm-1 receptor and its subsequent activity on key downstream signalling pathways. For example, in the perforated disks of temporomandibular joint (TMJ), angiogenesis appears to be regulated by factors that induce the NF-κB pathway [60]. Chm-1 expression was found to be significantly lower in perforated disks as compared to healthy disks, which appears to be regulated via the NF-κB pathway in the presence of interleukin-1β (IL-1β), linking Chm-1 to receptor-mediated NF-κB signaling and pathological angiogenesis of the TMJ [60]. Future studies to identify the specific receptor for Chm-1, and to determine the subsequent effect of signalling activation in a cell type dependent manner, are necessary to design therapeutic approaches for the treatment of Chm-1 related conditions, including osteoarthritis, infective endocarditis, and cancer.

## Summary and conclusion

**Fig. 5:**
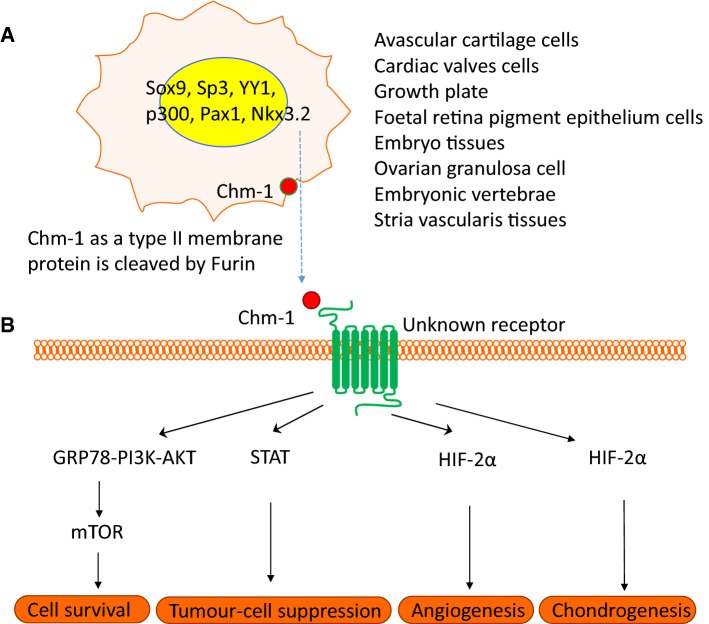
A hypothetical working model of Chm-1. **a** Chm-1 gene is expressed by many cell types and tissues such as avascular cartilage cells, cardiac valves, growth plate, foetal retina pigment epithelium cells, embryo tissue, ovarian granulosa cells, embryonic vertebrae, and stria vascularis tissues. Its expression is regulated by transcription factors including Sox9, Sp3, YY1, p300, Pax1, and Nkx3.2. **b** Precursor Chm-1, as a type II transmembrane glycoprotein protein is cleaved by furin to release its mature Chm-1. Mature Chm-1 binds to an unknown receptor in a cell type dependent manner and mediates downstream signalling cascades such as GRP78-PI3K-AKT and mTOR signalling for cell survival, Stat signalling for tumour cell suppression, and HIF-2α signalling for angiogenesis and chondrogenesis

Expression profiling indicates that Chm-1 is predominantly expressed in healthy and developing avascular cartilage, particularly during endochondral ossification, and in healthy cardiac valves (Fig. 5). Chm-1 plays a key role in chondrogenesis, chondrocyte maturation, and cartilage homeostasis. Decreased Chm-1 activity is implicated in the onset and progression of diseases, including osteoarthritis, infective endocarditis, and cancers. The anti-angiogenic and immune regulatory properties of Chm-1 are attributed to its role in disease prevention. The expression of Chm-1 appears to be regulated by several transcription factors, including Sox9, Sp3, YY1, p300, Pax1, and Nkx3.2. The Chm-1 receptor and downstream signalling effects remain largely unknown. Overall, Chm-1 represents a promising potential target for the delivery of therapeutic and bioengineering regenerative applications. Further research to elucidate the molecular mechanistic roadmap of Chm-1 will assist us to develop a therapeutic strategy for the treatment of diseases, such as osteoarthritis, infective endocarditis, and cancer.
